# ^18^F-THK5351 Positron Emission Tomography Imaging in Neurodegenerative Tauopathies

**DOI:** 10.3389/fnagi.2021.761010

**Published:** 2021-11-29

**Authors:** Michinori Ezura, Akio Kikuchi, Nobuyuki Okamura, Aiko Ishiki, Takafumi Hasegawa, Ryuichi Harada, Shoichi Watanuki, Yoshihito Funaki, Kotaro Hiraoka, Toru Baba, Naoto Sugeno, Shun Yoshida, Junpei Kobayashi, Michiko Kobayashi, Ohito Tano, Shun Ishiyama, Takaaki Nakamura, Ichiro Nakashima, Shunji Mugikura, Ren Iwata, Yasuyuki Taki, Katsutoshi Furukawa, Hiroyuki Arai, Shozo Furumoto, Manabu Tashiro, Kazuhiko Yanai, Yukitsuka Kudo, Atsushi Takeda, Masashi Aoki

**Affiliations:** ^1^Department of Neurology, Tohoku University Graduate School of Medicine, Sendai, Japan; ^2^Department of Occupational Therapy, Yamagata Prefectural University of Health Sciences, Yamagata, Japan; ^3^Division of Pharmacology, Faculty of Medicine, Tohoku Medical and Pharmaceutical University, Sendai, Japan; ^4^Department of Pharmacology, Tohoku University Graduate School of Medicine, Sendai, Japan; ^5^Department of Geriatrics and Gerontology, Institute of Development, Aging and Cancer, Tohoku University, Sendai, Japan; ^6^Division of Community of Medicine, Tohoku Medical and Pharmaceutical University, Sendai, Japan; ^7^Division of Cyclotron Nuclear Medicine, Cyclotron and Radioisotope Center, Tohoku University, Sendai, Japan; ^8^Division of Radiopharmaceutical Chemistry, Cyclotron and Radioisotope Center, Tohoku University, Sendai, Japan; ^9^Department of Neurology, National Hospital Organization Sendai Nishitaga Hospital, Sendai, Japan; ^10^Division of Neurology, Tohoku Medical and Pharmaceutical University, Sendai, Japan; ^11^Department of Diagnostic Radiology, Tohoku University Graduate School of Medicine, Sendai, Japan; ^12^Department of Nuclear Medicine and Radiology, Institute of Development, Aging and Cancer, Tohoku University, Sendai, Japan

**Keywords:** tau deposits, ^18^F-THK5351, positron emission tomography (PET), corticobasal syndrome (CBS), Alzheimer’s disease (AD), tauopathy, progressive supranuclear palsy (PSP), monoamine oxidase B (MAO-B)

## Abstract

**Introduction:** We aimed to determine whether *in vivo* tau deposits and monoamine oxidase B (MAO-B) detection using ^18^F-THK5351 positron emission tomography (PET) can assist in the differential distribution in patients with corticobasal syndrome (CBS), progressive supranuclear palsy (PSP), and Alzheimer’s disease (AD) and whether ^18^F-THK5351 retention of lesion sites in CBS and PSP can correlate with clinical parameters.

**Methods:** ^18^F-THK5351 PET was performed in 35 participants, including 7, 9, and 10 patients with CBS, PSP, and AD, respectively, and 9 age-matched normal controls. In CBS and PSP, cognitive and motor functions were assessed using the Montreal Cognitive Assessment, Addenbrooke’s Cognitive Examination–Revised, and Frontal Assessment Battery, Unified Parkinson’s Disease Rating Scale Motor Score, and PSP Rating Scale.

**Results:** ^18^F-THK5351 retention was observed in sites susceptible to disease-related pathologies in CBS, PSP, and AD. ^18^F-THK5351 uptake in the precentral gyrus clearly differentiated patients with CBS from those with PSP and AD. Furthermore, ^18^F-THK5351 uptake in the inferior temporal gyrus clearly differentiated patients with AD from those with CBS and PSP. Regional ^18^F-THK5351 retention was associated with the cognitive function in CBS and PSP.

**Conclusion:** Measurement of the tau deposits and MAO-B density in the brain using ^18^F-THK5351 may be helpful for the differential diagnosis of tauopathies and for understanding disease stages.

## Introduction

Tauopathies are a class of neurodegenerative diseases associated with the pathological aggregation of the tau protein in the brain. Progressive supranuclear palsy (PSP), corticobasal degeneration (CBD), and Alzheimer’s disease (AD) are common tauopathies. Typical pathological characteristics of PSP include tufted astrocytes, coiled bodies, and globose tangles. Conversely, CBD is pathologically characterized by astrocytic plaques and achromatic and ballooned neurons. Both PSP and CBD are four-repeat tauopathies and show overlapping pathological characteristics. Ultrastructurally, tau proteins in PSP and CBD form straight filaments, while those in AD form paired helical filaments. The clinical characteristics of corticobasal syndrome (CBS) include asymmetric motor symptoms (i.e., Parkinsonism, dystonia, and myoclonus) and cortical dysfunction. The CBS phenotypes include CBD, PSP, and AD. Because of the overlapping clinical symptoms of these diseases, their differential diagnosis is often difficult (Litvan et al., 2003 Osaki et al., 2004; Day et al., 2017).

Positron emission tomography (PET) has been used for the visualization of abnormal protein deposits such as amyloid-β (Klunk et al., 2004), tau, and α-synuclein (Kikuchi et al., 2010). The first generation of tau probes include ^11^C-PBB3 (Maruyama et al., 2013), ^18^F-AV-1451 (^18^F-T807) (Chien et al., 2013; Johnson et al., 2016; Smith et al., 2017a,b), and ^18^F-THK5351 (Harada et al., 2016; Kikuchi et al., 2016; Brendel et al., 2017; Ishiki et al., 2017; Okamura et al., 2018; Ezura et al., 2019). ^18^F-THK5351 (Harada et al., 2016 Brendel et al., 2017; Ishiki et al., 2017) was originally developed to visualize tau deposits in the AD brain. However, ^18^F-THK5351 shows high binding affinity to monoamine oxidase B (MAO-B), which enables astrocytosis visualization in the human brain (Harada et al., 2018; Ishiki et al., 2018; Tago et al., 2019). MAO-B inhibitor prescription has reduced ^18^F-THK5351 retention on average by 36.7–51.8% (Ng et al., 2017). In several neurodegenerative diseases, one of the prominent changes is reactive astrogliosis associated with the accumulation of misfolded proteins (Leyns and Holtzman, 2017). Therefore, the spatial information of ^18^F-THK5351 binding is expected to be useful for the early and differential diagnosis of tauopathies and disease progression monitoring. Tau PET studies have been performed in patients with CBS, PSP, and AD (Dani et al., 2016; Coakeley and Strafella, 2017). However, comparative study of these three tauopathies have never been performed at a single center. In the present study, we aim to evaluate the clinical utility of tau deposits and MAO-B PET imaging using ^18^F-THK5351 for the differential diagnosis and clinical assessment of tauopathies.

## Materials and Methods

### Participants

A total of 35 participants were included in this study: seven patients with probable CBS (Supplementary Table 1), nine with probable PSP (Richardson syndrome type), and 10 with probable AD and 9 age-matched normal controls (NCs) (Table 1). All of them underwent ^18^F-THK5351 PET scans. The diagnoses of CBS, PSP, and AD were made based on the modified Cambridge criteria (Mathew et al., 2012), National Institute of Neurological Disorders and Stroke and the Society for PSP (NINDS-SPSP) criteria (Litvan et al., 1996), and National Institute of Neurological and Communicative Disorders and Stroke and the Alzheimer’s Disease and Related Disorders Association (NINCDS-ADRDA) criteria (McKhann et al., 1984), respectively. The Mini-Mental State Examination (MMSE), Montreal Cognitive Assessment (MoCA), Addenbrooke’s Cognitive Examination–Revised (ACE-R), and Frontal Assessment Battery (FAB) were used to assess the cognitive performance of the participants with CBS and PSP. However, one patient with PSP could not perform the ACE-R and MoCA. Furthermore, the motor functions were assessed using the Unified Parkinson’s Disease Rating Scale (UPDRS) motor score and Progressive Supranuclear Palsy Rating Scale (PSPRS) in CBS and PSP. The NC group was comprised of volunteers with no cognitive or motor function impairments nor any observable cerebrovascular lesions through MRI scans. This study protocol was approved by the Ethics Committee of Tohoku University Hospital (approval number, 2016-2-266) and registered to the UMIN Clinical Trials Registry (registration number, UMIN000021819). Written informed consent was obtained from each patient or his/her guardian(s) after they were given a complete description of the study.

**TABLE 1:** Demographic characteristics of CBS, PSP, AD, and NC participants.

	CBS		PSP	AD	NC
Number (M/F)	7 (2/5)		9 (9/0)	10 (7/3)	9 (5/4)
**Age (years)**					
Mean	69.1 ± 4.85		74.1 ± 5.28	75.6 ± 10.0	71.0 ± 6.44
Range	64–78		66–81	58–88	61–81
Education (years)	11.6 ± 1.13		14.1 ± 2.26	12.4 ± 2.41	12.9 ± 2.52
11C-PiB SUVR in neocort	1.18 ± 0.11#		1.20 ± 0.12#	1.96 ± 0.28	1.14 ± 0.09 #
**Cognitive scores**					
MMSE score	23.3 ± 6.85		21.8 ± 6.63*	18.9 ± 4.65**	28.8 ± 1.56
MoCA score	18.0 ± 8.41		14.9 ± 4.73	NE	NE
ACE-R score	66.7 ± 27.7		63.9 ± 19.3	NE	NE
FAB score	9.90 ± 4.91		9.00 ± 4.06	NE	NE
**Motor scores**					
UPDRS motor score	33.7 ± 18.3		38.9 ± 14.6	NE	NE
PSPRS score	34.0 ± 21.7		44.9 ± 19.4	NE	NE
**Medication (mg/day)**					
L-dopa	191 ± 269		500 ± 249	0	0
Selegiline		0	0	0	0
Amantadine	0		38.9 ± 78.2	20.0 ± 63.2	0
Donepezil	0.71 ± 1.89		0.56 ± 1.67	5.50 ± 4.38	0
Galantamine	0		0	3.20 ± 6.75	0
Memantine	0		1.11 ± 3.33	10.0 ± 10.5	0

*The data are expressed as the mean ± standard deviation.*

*#p < 0.001 comparing with AD by the one-way ANOVA for multiple comparisons.*

*p < 0.05 and **p < 0.01 comparing with NC by the one-way ANOVA for multiple comparisons.

*ACE-R, Addenbrooke’s cognitive examination–revised; AD, Alzheimer’s disease; CBS, corticobasal syndrome; FAB, frontal assessment battery; MMSE, mini-mental state examination; MoCA, Montreal cognitive assessment; NC, normal control; NE, not examined; PiB, Pittsburgh compound B; PSP, progressive supranuclear palsy; PSPRS, progressive supranuclear palsy rating scale; UPDRS, unified Parkinson’s disease rating scale.*

### Image Acquisition

^18^F-THK5351 and ^11^C-Pittsburgh compound B (PiB) were manufactured at the Cyclotron and Radioisotope Center of Tohoku University using a semiautomated system developed in-house. The injectable solutions of ^18^F-THK5351 and ^11^C-PiB were obtained with a radiochemical purity of >95% and specific activity of 357 ± 270 and 240 ± 48 GBq/μmol. PET imaging was performed using an Eminence STARGATE scanner (Shimadzu, Kyoto, Japan). After rapidly injecting 185-MBq ^18^F-THK5351 or 296-MBq ^11^C-PiB, PET images were obtained for 20 min (4 scans × 300 s) from 40 to 60 min or 50–70 min postinjection and used for further analysis. In addition, a 3D volumetric acquisition of a T1-weighted gradient echo sequence produced a gapless series of thin axial sections using a spoiled gradient recall sequence (echo time/repetition time, 3.4/7.2 ms; flip angle, 9°; acquisition matrix, 240 × 256; 1 excitation; field of view, 25.6 cm; slice thickness, 0.8 mm) of a Vantage Titan™ 3T scanner (Canon Medical Systems, Otawara, Japan).

### Image Analysis

The standardized uptake value (SUV) images of ^18^F-THK5351 and ^11^C-PiB were obtained by normalizing the tissue radioactivity concentration to the injected dose and body weight. The PNEURO module in PMOD software (version 3.6; PMOD Technologies, Zurich, Switzerland) was used to place and evaluate the volumes of interest (VOIs) through an automatic VOI method. The PET images were matched rigidly to the T1-weighted MR images acquired from each participant. Then, the MR images were spatially normalized to the Montreal Neurological Institute T1-MRI template. The VOIs were automatically outlined on the normalized MR images based on the maximum probability atlas constructed by the automated anatomic labeling template used in the PMOD software. Subsequently, they were defined in the 13 regions; precentral, superior frontal, postcentral, and superior parietal gyri, parahippocampus, fusiform and inferior temporal gyri, putamen, pallidum, thalamus, midbrain, cerebral white matter, and cerebellar cortex. In a normal brain, the cerebellum is a region among those with the lowest MAO-B concentrations (Tong et al., 2013). Because tau deposition is usually minimal in the cerebellar cortex, increased MAO-B signals related to tau depositions would be expected to be negligible. Therefore, the regional-to-cerebellar-cortex SUV ratio (SUVR) was used as a tracer retention index.

Neocortical amyloid-β burden was expressed as the average SUVR for the following cortical VOIs: superior frontal, superior parietal, middle and inferior temporal, and posterior cingulate gyri for ^11^C-PiB. As in the previous studies (Ezura et al., 2019), an ^11^C-PiB SUVR threshold of 1.5 was used to categorize high and low amyloid-β burden.

### Statistical Analysis

The one-way ANOVA and Steel–Dwass test for multiple comparisons was used to identify the group differences in clinical variables (e.g., age, years of education, MMSE score, and ^11^C-PiB SUVR value) and assess the differences in the regional ^18^F-THK5351 SUVR values, respectively. Moreover, the group differences in clinical variables including MoCA, ACE-R, FAB, UPDRS motor, and PSPRS scores between CBS and PSP were identified using the Mann–Whitney *U* test. Pearson’s correlation coefficients were also calculated to assess the relationship between ^18^F-THK5351 SUVR values and MMSE, MoCA, ACE-R, FAB, UPDRS motor, and PSPRS scores without the correction for multiple comparisons. Moreover, statistical relations of ^18^F-THK5351 SUVR values and clinical parameters were calculated based on the region’s base and drawn into correlation matrices. The four aforementioned tests were performed using GraphPad Prism 6 software (GraphPad, San Diego, CA, United States). The data were expressed as the mean ± standard deviation.

## Results

### Clinical Characteristics of the Corticobasal Syndrome, Progressive Supranuclear Palsy, Alzheimer’s Disease, and Normal Control Participants

The clinical characteristics of all participants are summarized in Table 1. Among the four groups, no significant differences in the age and years of education were noted. However, the MMSE scores in the PSP and AD groups were significantly lower than that in the NC group. No significant differences in the MoCA, ACE-R, FAB, UPDRS motor, and PSPRS scores were noted between the CBS and PSP groups. Additionally, PiB-PET showed negative neocortical signals in all CBS, PSP, and NC subjects, while all of 10 patients with AD were positive. Therefore, all of CBS patients in this study are possibly CBS-CBD, although it is not pathologically proven.

### Clinical Positron Emission Tomography Studies

**FIGURE 1:**
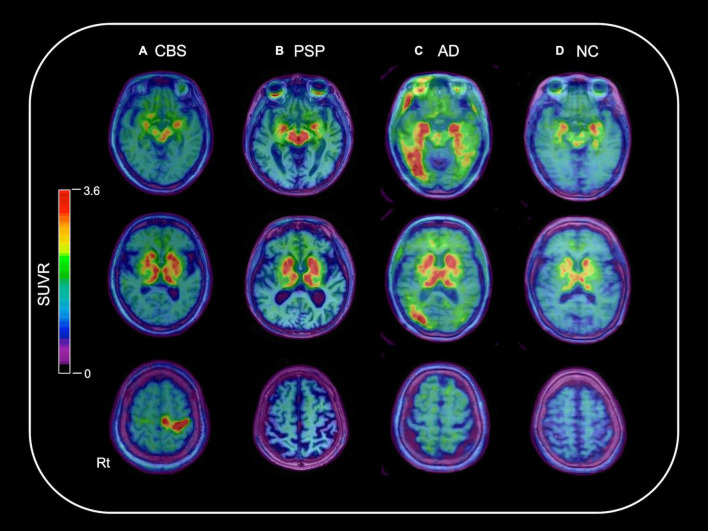
Representative ^18^F-THK5351 PET images overlaid on the baseline MRI data of the CBS, PSP, AD, and NC participants. A 70-year-old female with corticobasal syndrome (CBS) **(A)**, a 79-year-old male with progressive supranuclear palsy (PSP) **(B)**, and a 74-year-old female with Alzheimer’s disease (AD) **(C)** showed high ^18^F-THK5351 signals in the precentral and postcentral gyri; midbrain; and inferior temporal, fusiform, and parahippocampal gyri, respectively, compared with a 71-year-old female in the normal control (NC) group **(D)**.

Figure 1 shows the representative ^18^F-THK5351 PET images of the CBS, PSP, AD, and NC participants. High ^18^F-THK5351 signals were noted in the precentral and postcentral gyri of the patients with CBS, midbrain of the patients with PSP, and inferior temporal, fusiform, and parahippocampal gyri of the patients with AD compared with those of the NC participants. All patients with AD in this study showed typical subtype. AD patients showing hippocampal-sparing or limbic-predominant subtypes are not included in this study.

**FIGURE 2:**
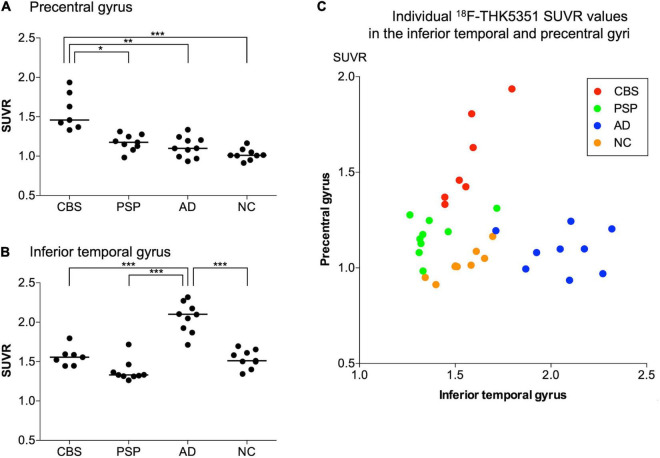
^18^F-THK5351 SUVR values in the precentral and inferior temporal gyri. There were significant differences in ^18^F-THK5351 SUVR values in the precentral gyrus of patients with corticobasal syndrome (CBS) compared with those in the progressive supranuclear palsy (PSP), Alzheimer’s disease (AD), and normal control (NC) groups **(A)** and in the inferior temporal gyrus of the patients with AD compared with those in the other groups **(B)**. The scatter chart showed ^18^F-THK5351 SUVR values in the inferior temporal (x-axis) and precentral (y-axis) gyri of the participants in four groups. The participants with CBS, PSP, AD, and NC are represented by red, green, blue, and orange dots, respectively. The individual bivariate values clearly discriminated the patients among three groups **(C)**. *p < 0.005, **p < 0.0005, and ***p < 0.0001 by Steel–Dwass test for multiple comparisons.

**FIGURE 3:**
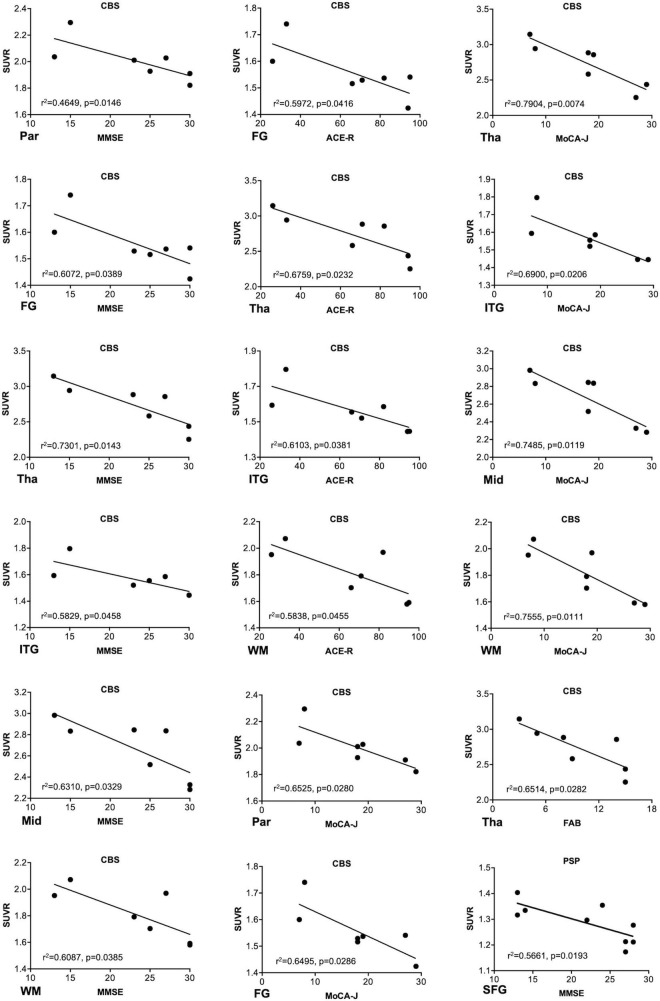
Associations between the regional^18^F-THK5351 retention and cognitive and motor function scores. The regional^18^F-THK5351 retention showed associations with the Mini-Mental State Examination (MMSE), Montreal Cognitive Assessment (MoCA), Addenbrooke’s Cognitive Examination–Revised (ACE-R), and Frontal Assessment Battery (FAB) scores in corticobasal syndrome (CBS) and MMSE score in progressive supranuclear palsy (PSP). FG, fusiform gyrus; ITG, inferior temporal gyrus; Mid, midbrain; Par, parahippocampus; SFG, superior frontal gyrus (dorsolateral); Tha, thalamus; WM, cerebral white matter. *P* values were uncorrected because of no correction for multiple comparisons.

^18^F-THK5351 SUVR values in the four groups are shown in Table 2. In the patients with CBS, ^18^F-THK5351 retention was significantly greater in the precentral gyrus compared with those in the PSP, AD, and NC groups (Figure 2A). This retention was also significantly greater in the inferior temporal and fusiform gyri of the patients with AD than those in the other groups (Figure 2B). The individual bivariate ^18^F-THK5351 SUVR values in the precentral and inferior temporal gyri clearly show the discrimination among three groups (Figure 2C). In the patients with CBS and PSP, associations were noted between cognitive performances and spatial distribution of ^18^F-THK5351 signals (Figure 3). Supplementary Table 2 shows the result of correlation matrices.

**TABLE 2:** Regional ^18^F-THK5351 SUVR values in CBS, PSP, AD, and NC participants.

Region (abbreviation)	CBS	PSP	AD	NC
Precentral gyrus (PreG)	1.57 ± 0.23^Pb,^ ^Ad,^ ^Ne^	1.17 ± 0.10^Cb^	1.12 ± 0.13^Cd^	1.02 ± 0.07^Ce^
Superior frontal gyrus, dorsolateral (SFG)	1.47 ± 0.15^Na^	1.29 ± 0.08	1.36 ± 0.13	1.16 ± 0.12^Ca^
Postcentral gyrus (PosG)	1.42 ± 0.23^Na^	1.17 ± 0.12	1.21 ± 0.12	1.10 ± 0.08*^Ca^*
Superior parietal gyrus (SPG)	1.40 ± 0.28^Na^	1.14 ± 0.13	1.29 ± 0.22	1.04 ± 0.11*^Ca^*
Parahippocampus (Par)	2.00 ± 0.15^Ac^	1.82 ± 0.17^Ae^	2.43 ± 0.20^Cc,^ ^Pe,^ ^Nd^	2.01 ± 0.12^Ad^
Fusiform gyrus (FG)	1.56 ± 0.10^Ae^	1.41 ± 0.14^Ae^	2.06 ± 0.19^Ce,^ ^Pe,^ ^Ne^	1.60 ± 0.11^Ae^
Inferior temporal gyrus (ITG)	1.56 ± 0.12^Ae^	1.38 ± 0.14^Ae^	2.11 ± 0.25^Ce,^ ^Pe,^ ^Ne^	1.53 ± 0.12^Ae^
Putamen (Put)	2.92 ± 0.37^Pa,^ ^Aa,^ ^Nc^	2.60 ± 0.29^Ca^	2.60 ± 0.34^Ca^	2.48 ± 0.30^Cc^
Pallidum (Pal)	3.92 ± 0.61^Pa,^ ^Ae,^ ^Ne^	3.57 ± 0.41^Ca^ ^Aa,^ ^Nc^	3.28 ± 0.43^Ce,^ ^Pa^	3.16 ± 0.41^Ce,^ ^Pc^
Thalamus (Tha)	2.73 ± 0.31^Pa,^ ^Ae,^ ^Nb^	2.38 ± 0.26^Ca^	2.17 ± 0.20^Ce^	2.32 ± 0.20^Cb^
Midbrain (Mid)	2.66 ± 0.28^Ae,^ ^Nc^	2.41 ± 0.14^Aa^	2.09 ± 0.24^Ce,^ ^Pa^	2.22 ± 0.15^Cc^
Cerebral white matter (WM)	1.81 ± 0.1^Na^	1.61 ± 0.10	1.69 ± 0.11	1.50 ± 0.13*^Ca^*

*Data are mean ± standard deviation.*

*^a^p < 0.05, ^b^p < 0.005, ^c^p < 0.001, ^d^p < 0.0005, and ^e^p < 0.0001 by Steel–Dwass test for multiple comparisons.*

*^C^versus CBS, ^P^versus PSP, ^A^versus AD, ^N^versus NC by Steel–Dwass test for multiple comparisons.*

*AD, Alzheimer’s disease; CBS, corticobasal syndrome; NC, normal control; PSP, progressive supranuclear palsy; SUVR, standardized uptake value ratio.*

## Discussion

Clinical and pathological overlaps between CBS and PSP (Armstrong et al., 2013) make the differential diagnosis of CBS and PSP difficult. Thus, several studies have been conducted to explore novel neuroimaging markers (Niethammer et al., 2014; Fujioka et al., 2021). ^18^F-THK5351 PET showed the characteristic spatial pattern of tracer distribution in CBS, PSP, and AD. High ^18^F-THK5351 uptake was observed in the precentral and postcentral gyri; midbrain; and inferior temporal, fusiform, and parahippocampal gyri of the patients with CBS, PSP, and AD, respectively (Figure 1). These spatial patterns of ^18^F-THK5351 retention were consistent with the distribution of gliosis in the postmortem analysis of the brains of the patients with CBD, PSP, and AD (Yoshida, 2014; Harada et al., 2018). The precentral gyrus, where patients with CBS showed the highest retention of ^18^F-THK5351, was the best region for the differentiation between CBS and PSP or AD (Figure 2A). Furthermore, the inferior temporal gyrus, where a high density of paired helical filament-tau and associated gliosis have been observed in AD (Harada et al., 2018), was a good region to differentiate AD from PSP and CBS, because patients with AD showed higher retention of ^18^F-THK5351 in the region compared to those with CBS and PSP (Figure 2B). Thus, the bivariate ^18^F-THK5351 SUVR values in these two regions clearly distinguished the patients among three groups (Figure 2C). Our results suggest that tau deposits and MAO-B imaging by ^18^F-THK5351 PET is a valuable method for the differential diagnosis of tauopathies. Moreover, one of the advantages of this study is the consistent acquisition of PET images in CBS, PSP, and AD patients in a single center, which minimizes data variability.

In this study, we additionally evaluated the relationship between the clinical parameters and SUVR values of ^18^F-THK5351. ^18^F-THK5351 retention was associated with cognitive parameters in the patients with CBS and PSP (Figure 3), while no significant association was observed between ^18^F-THK5351 retention and motor parameters. These results differed from the previous results in PSP (Brendel et al., 2017) and in CBS (Kikuchi et al., 2016). In the prior study, significant relation between the clinical motor severity and ^18^F-THK5351 retention in midbrain was demonstrated in the patients with PSP (Brendel et al., 2017). Contrarily, all of patients with PSP in the present study met the criteria for the Richardson syndrome type and were administrated with L-dopa (500 ± 249 mg). Supposedly, dopamine-replenishing therapy may somehow alleviate motor dysfunction in PSP and obscure the relationship between the clinical motor and imaging parameters. Further research is needed to clarify the correlations between clinical motor severities and ^18^F-THK5351 retention in more patients with PSP with various subtypes and stages. On the other hand, our previous report failed to elucidate a meaningful relationship between clinical severities including cognitive dysfunction and ^18^F-THK5351 retention in patients with CBS because of the very limited small sample size (Kikuchi et al., 2016). ^18^F-THK5351 PET may be useful to evaluate disease stages in CBS.

Several limitations are noted in this study. Firstly, our sample size was relatively small. Secondly, there was the absence of neuropathological confirmation of clinical diagnosis for all the cases. Thirdly, the current PET data are difficult to interpret due to the non-selective binding properties of ^18^F-THK5351 to MAO-B and tau. However, because of the substantial reduction of ^18^F-THK5351 retention after the treatment with the MAO-B inhibitor (Ng et al., 2017), we consider that a majority of the tracer signals in ^18^F-THK5351 PET are derived from the binding to MAO-B (Ishiki et al., 2018). The elevation of MAO-B level is thought to reflect reactive astrogliosis caused by tau burden in the affected brain regions. Thus, ^18^F-THK5351 signals reflect a sequence of pathogenic process in tauopathies. In the future, selective PET tracers for MAO-B or four-repeat tau will clarify the association of tau protein accumulation and reactive astrocytosis in the PSP and CBD pathogenesis.

In conclusion, the spatial pattern of ^18^F-THK5351 PET signals adequately coincided with the disease-related regions and was a good diagnostic marker in neurodegenerative tauopathies. Considering the high binding affinity of THK5351 to tau deposits and MAO-B, ^18^F-THK5351 PET imaging is a potentially useful technique in the differential diagnosis of tauopathies.

## Data Availability Statement

The raw data supporting the conclusions of this article will be made available by the authors, without undue reservation.

## Ethics Statement

The studies involving human participants were reviewed and approved by the Ethics Committee of Tohoku University Hospital (approval number, 2016-2-266). The patients/participants provided their written informed consent to participate in this study.

## Author Contributions

AK and NO designed the experiments. ME, AK, and NO analyzed and interpreted the data. ME, AK, NO, and TH wrote the manuscript. All authors contributed to the data collection and analysis and reviewed the manuscript.

## Conflict of Interest

NO, SF, and YK declare that this study received funding from GE Healthcare, Clino Ltd. and Sumitomo Electric Industries. The funder was not involved in the study design, collection, analysis, interpretation of data, the writing of this article or the decision to submit it for publication.

## Publisher’s Note

All claims expressed in this article are solely those of the authors and do not necessarily represent those of their affiliated organizations, or those of the publisher, the editors and the reviewers. Any product that may be evaluated in this article, or claim that may be made by its manufacturer, is not guaranteed or endorsed by the publisher.
